# Screening for atrial fibrillation with baseline and intermittent ECG recording in an out-of-hospital population

**DOI:** 10.1186/1471-2261-13-41

**Published:** 2013-06-10

**Authors:** Tijn Hendrikx, Rolf Hörnsten, Mårten Rosenqvist, Herbert Sandström

**Affiliations:** 1 Department of Public Health and Clinical Medicine, Family Medicine, Umeå University, Umeå, SE-901 87, Sweden; 2 Clinical Physiology, Heart Centre and Department of Surgical and Perioperative Science, Umeå University, Umeå, SE-901 87, Sweden; 3 Department of Clinical Science, Karolinska Institutet, Stockholm, Danderyds Sjukhus, SE-182 88, Sweden

**Keywords:** Arrhythmia, Atrial fibrillation, Handheld ECG, Stroke prevention, Screening

## Abstract

**Background:**

the objective of this study is to investigate the detection rate of undiagnosed atrial fibrillation (AF) with short intermittent ECG recordings during four weeks among out-of-hospital patients, having at least one additional risk factor (CHADS_2_) for stroke.

**Method:**

*Design*: Cross-sectional study. *Setting*: Eight family practice centres and two hospital-based out-patient clinics in Sweden. *Subjects*: 989 out-of-hospital patients, without known AF, having one or more risk factors associated with stroke (CHADS_2_). *Interventions*: All individuals were asked to perform 10-second handheld ECG recordings during 28 days, twice daily and when having palpitations. *Main outcome measures*: Episodes of AF on handheld ECG recordings were defined as irregular supraventricular extrasystoles in series with a duration of 10 seconds.

**Results:**

928 patients completed registration. AF was found in 35 of 928 patients; 3.8% (95% confidence interval [CI] 2.7–5.2). These 35 patients had a mean age of 70.7 years (SD ± 7.7; range 53–85) and a median CHADS_2_ of 2 (range 1–4).

**Conclusions:**

Intermittent handheld ECG recording over a four week period had a detection rate of 3.8% newly diagnosed AF, in a population of 928 out-of-hospital patients having at least one additional risk factor for stroke. Intermittent handheld ECG registration is a feasible method to detect AF in patients with an increased risk of stroke in whom oral anticoagulation (OAC) treatment is indicated.

## Background

Atrial fibrillation/flutter (AF) is the most common type of arrhythmia occurring in 1–2% of the adult population. With increasing age the prevalence of AF increases. Approximately 5% of the population over 65 years and 10% of the population over 80 years has AF [1-5]. A recent Swedish study shows an even higher overall prevalence of 2.5% and a prevalence of 13.8% in those over 80 years [6].

Occurrence of AF constitutes in itself an independent risk factor for stroke [7], and with other concurrent risk factors, congestive heart failure, hypertension, age ≥75 years, diabetes and earlier stroke (CHADS_2_ score), this risk is additionally increased [8,9]. In AF patients with a CHADS_2_ score of ≥ 1, treatment with oral anticoagulation (OAC) should be considered. In AF patients with a CHADS_2_ score of ≥ 2 chronic OAC is recommended to prevent stroke [9].

AF was found in 28.7% (of 152 746) Swedish stroke patients at admission to hospital 2001–2008 [10]. Since AF is often asymptomatic it may remain undiagnosed for a long time and many of these patients will not receive OAC before suffering a stroke [11]. It has been estimated that only one in ten paroxysms of AF are symptomatic [12,13]. Several studies have shown that patients with paroxysmal AF have the same stroke incidence as patients with permanent AF [14-16].

It has been estimated that up to 8% of stroke patients without known AF have episodes of asymptomatic AF beyond that detected by physical examination and initial ECG during hospital admission for acute ischemic stroke [17-19]. A recent study shows that intermittent handheld ECG recording after carrying out Holter ECG substantially improves the detection of asymptomatic atrial fibrillation (AF) in post-stroke patients [20].

AF in the general population is traditionally detected with pulse registration/palpation, 12-lead resting ECG [21-23] or 24-hour Holter/Event recorder ECG [24], but these methods have a relatively low sensitivity for detection of asymptomatic AF. As a result the prevalence of undiagnosed AF in the general population is not well known.

The objective of this study is to estimate the detection rate of undiagnosed AF among out-of-hospital patients, having at least one additional risk factor (CHADS_2_) for stroke, using intermittent 10-second ECG recording during 28 days with a handheld device.

## Methods

### Design, study population and setting

In this cross-sectional study patients from eight family practice centres and two hospital-based out-patient clinics in Sweden, having one or more risk factors associated with stroke (CHADS_2_ score), without known AF, were identified from physicians’ and nurses’ surgery lists and included consecutively.

### Inclusion period

The inclusion period extended from May 2007 until June 2011.

### Intervention

Inclusion criteria: one or more risk factors associated with stroke (CHADS_2_ score). Exclusion criteria: known AF, impaired cognitive function or other functional impairments that prevent the use of the handheld device. When AF was detected during the study an additional review of the patient’s medical record and existing ECGs five years back in time was done to reveal unreported previous episodes of AF. Patients with such episodes of AF were excluded.

**Figure 1:**
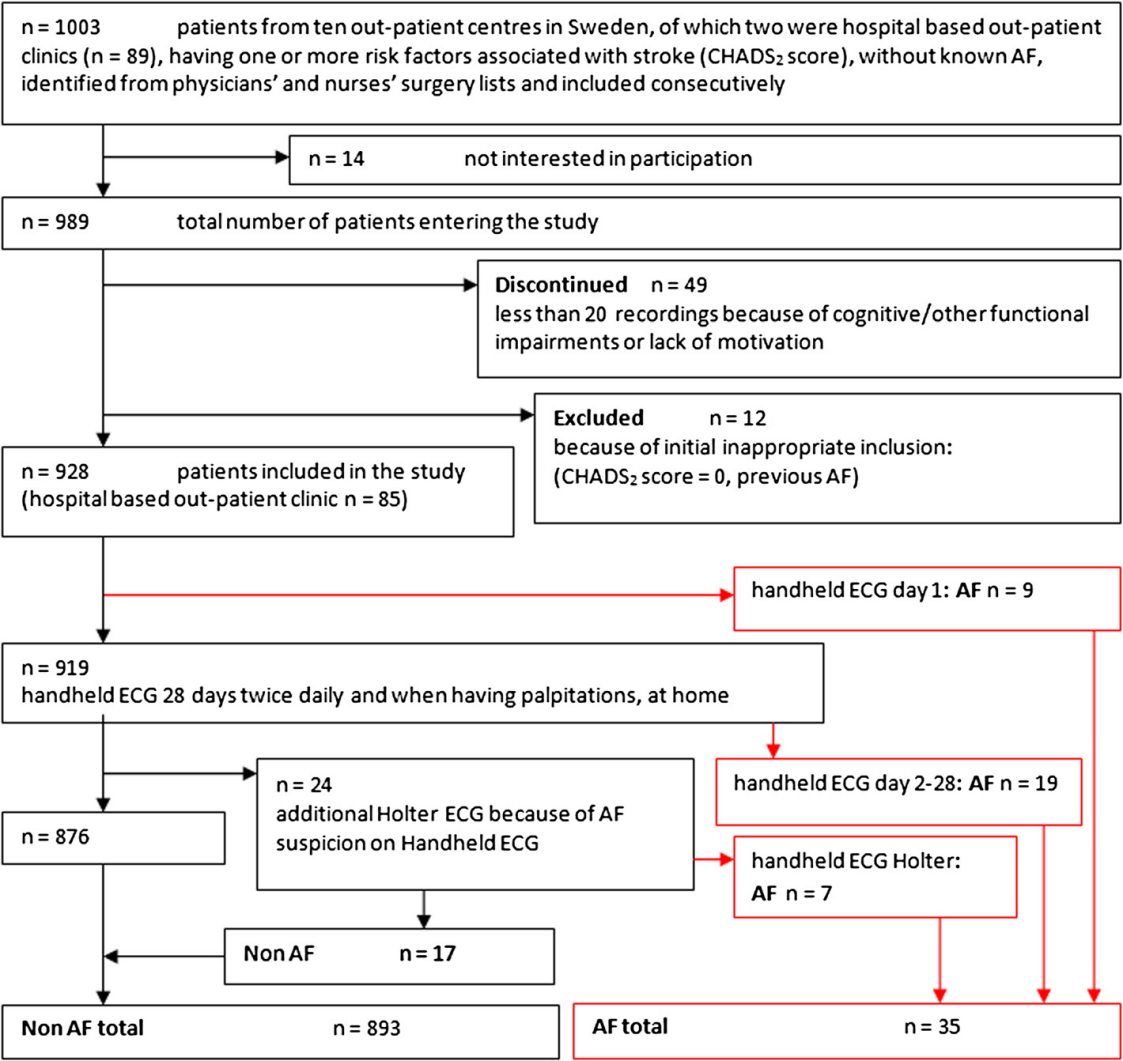
Study flowchart.

After giving informed consent patients were instructed to perform a 10 second handheld ECG (Zenicor EKG^®^). When AF was detected at the first registration, patients were referred for treatment without further registrations. If no AF was seen at the first registration, patients were asked to perform a similar 10-second handheld ECG (Zenicor EKG^®^) registration during 28 days, twice daily (morning and evening) and when having palpitations. After every registration the recording was transmitted by the patient via phone to an internet-based central database. ECG registrations were evaluated at least once a week by a study nurse and when in doubt the ECG was additionally checked by a single physician, having the possibility to contact a cardiologist in uncertain cases. Patients with detected AF were referred for treatment in accordance with national guidelines to the patients’ regular family physician or to a cardiologist. Patients with less than 20 registrations were excluded. (For additional information see Figure 1: study flowchart).

### Outcome measures

We defined episodes of AF on handheld ECG recordings as irregular supraventricular extrasystoles in series with a duration of 10 seconds. Ambiguous recordings, showing repetitive irregular supraventricular extrasystoles (SVES) less than 10 seconds, were referred for an additional 24-hour Holter ECG. AF on Holter was defined by at least 10 seconds showing irregular rhythm without sinus P-waves.

Paroxysmal AF was defined as self-terminating, usually within seven days. Persistent AF was defined as an AF episode that either lasts longer than 7 days (but less than a year) or requires termination by cardioversion, either with drugs or by direct current cardioversion (DCC) [9].

### The device

**Figure 2:**
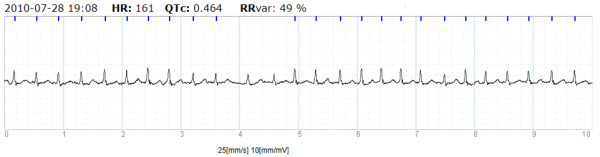
Example of registration of AF with handheld ECG.

**Figure 3:**
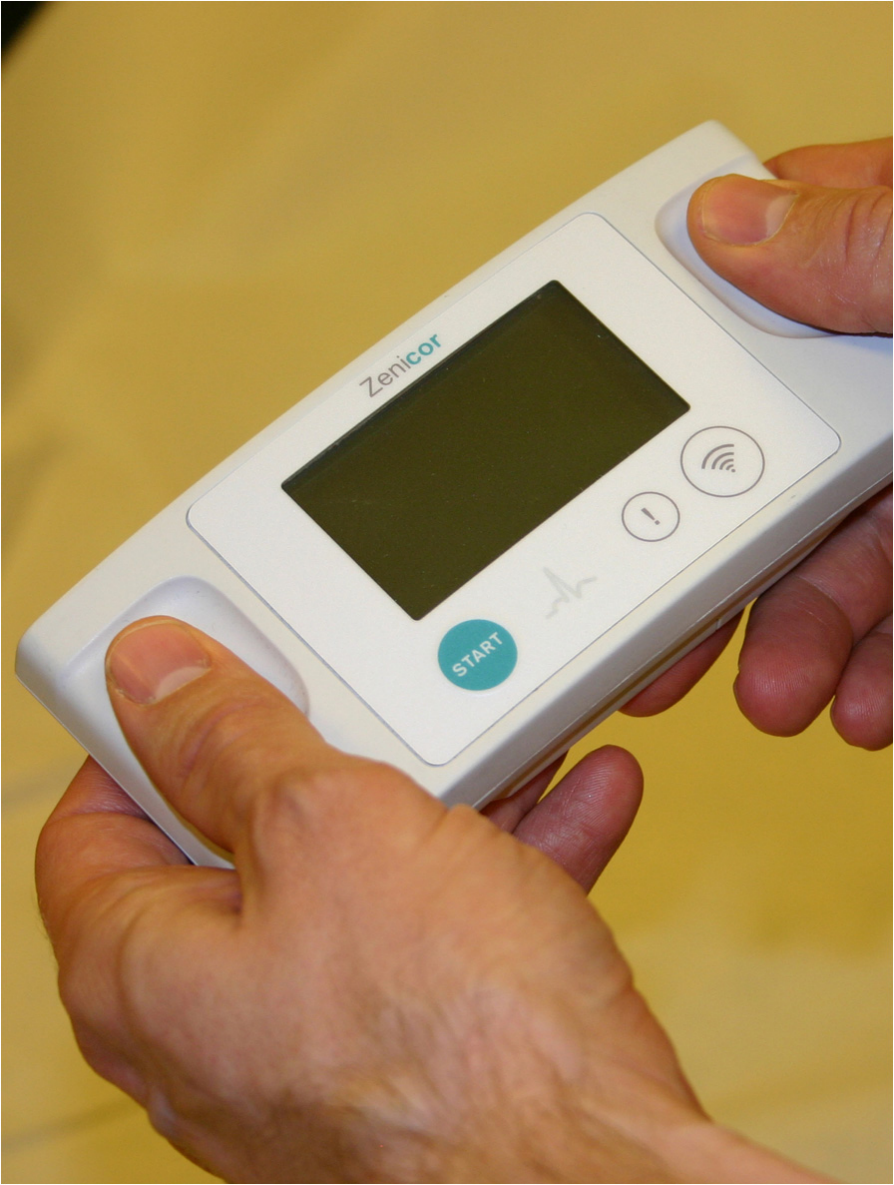
Photo of the handheld ECG device.

ECG recordings were performed using a handheld device, Zenicor EKG^®^, which via both thumbs registers a bipolar extremity lead I ECG during 10 seconds. After each registration the recording was transmitted by the patient via phone to a web-based central database. The ability to give the correct diagnosis of AF compared to 12-lead ECG has shown a sensitivity of 96% and a specificity of 92% [25]. A detailed technical description of the device, and its performance is published elsewhere [25]. (An example of AF as recorded with the handheld ECG device is given in Figure 2. Figure 3 shows a photo of the handheld ECG device).

### Statistics

The statistics of this study were mainly descriptive. Continuous variables were presented with mean, standard deviation (SD) and range (minimum and maximum) whereas categorical variables were presented with count and percentage and, where appropriate, a 95% confidence interval. Pearson Chi Square test was used to test for differences in AF detection rate for gender, age categories and CHADS_2_ risk factors. To evaluate possible differences in the number of registrations, age and total CHADS_2_ risk score between individuals with and without detected AF, the Mann–Whitney U test was used. SPSS Statistics 19.0 (IBM Corporation, Route 100 Somer, NY 10589) was used for all calculations. The level of significance was set at 0.05, two-sided. The sample size was based on the hypothesis of finding 4% AF. Given approximately 1000 patients this would yield a 95% confidence interval of ± 1%, which was deemed narrow enough to answer the objective.

### Ethical considerations

The study was approved by the Regional Ethics Committee (Dnr 07-051 M). All participating patients gave written informed consent.

## Results

### Demographics

A total of 989 patients, 491 men and 498 women, entered the study. Forty-nine patients with fewer than 20 recordings did not complete registration because of technical or medical problems. Twelve patients were excluded due to initial inappropriate inclusion. Clinical characteristics of the remaining 928 patients are displayed in Table 1. The mean number of registrations per patients, with respect to a recommended number of 56 registrations, was 55.4 registrations (SD ± 11.2 and range 1–125). There was no statistical correlation between AF detection and the number of registrations (Mann–Whitney U test, P = 0.442).

**Table 1:** **Characteristics of the total study population**, **AF patients and non**-**AF patients**

	**Total population**			**AF**			**Non-AF**			**P-value**
					*detection rate (%)*	*95% CI*				
**Total, n**	928			35	3.8%	(2.7–5.2)	893			
**Men, n (%)**	462 (49.8%)			21 (60.0%)	4.6%	(3.0–6.8)	441 (49.4%)			0.218
**Women, n (%)**	466 (50.2%)			14 (40.0%)	3.0%	(1.8–5.0)	452 (50.6%)			
**Age < 65 years, n (%)**	242 (26.1%)			7 (20.0%)	2.9%	(1. 4–5.8)	235 (26.3%)			0.404
**Age ≥65 years, n (%)**	686 (73.9%)			28 (80.0%)	4.1%	(2.8–5.8)	658 (73.7%)			
**CHADS2 = 1, n (%)**	415 (44.7%)			13 (37.1%)	3.1%	(1.7–5.1)	403 (45.1%)			0.351
**CHADS2 ≥ 2, n (%)**	513 (55.3%)			22 (62.9%)	4.3%	(2.8–6.3)	490 (54.9%)			
**CHF, n (%)**	34 (3.7%)			1 (2.9%)	2.9%	(0.7–14.9)	33 (3.7%)			0.796
**HT, n (%)**	838 (90.3%)			31 (88.6%)	3.7%	(2.6–5.2)	807 (90.4%)			0.724
**Age ≥75 y, n (%)**	326 (35.0%)			13 (37.1%)	4.0%	(2.6–7.1)	313 (35.1%)			0.799
**DM, n (%)**	293 (31.6%)			12 (34.3%)	4.1%	(2.4–7.0)	281 (31.5%)			0.725
**ES, n (%)**	80 (8.6%)			4 (11.4%)	5.0%	(2.0–12.2)	76 (8.5%)			0.546
IHD*, n (%)	184 (19.8%)			5 (14.3%)	2.7%	(1.2–6.2)	179 (20.0%)			0.402
	**Total population**			**AF**			**Non-AF**			
	***Mean***	***SD***	***Range***	***Mean***	***SD***	***Range***	***Mean***	***SD***	***Range***	***P-value***
**Number of ECG registrations, n**	55.4	± 11.2	1–125	50.3	± 22.1	1–96	55.6	± 10.5	20–125	0.442
**Age, y**	69.8	± 9.4	33–89	70.7	± 7.7	53–85	69.8	± 9.4	33–89	0.679
	***Median***		***Range***	***Median***		***Range***	***Median***		***Range***	
**CHADS**_**2 **_**total**	2		1–5	2		1–4	2		1–5	0.451

*CHF *Congestive heart failure, *HT *Hypertension, *DM* Diabetes mellitus, *ES *Earlier stroke/TIA, *IHD *Ischemic heart disease (* not included in CHADS2). P-values compare AF with non-AF patients. Participants can have more than one co-morbidity.

### Detection of AF and AF characteristics

Analysis of our data showed newly diagnosed AF in 35 of these 928 patients (3.8% (95% confidence interval [CI] 2.7–5.2)). Characteristics of AF patients are displayed in Table 1 and a study flowchart (Figure 1). Twenty-eight of these AF patients were detected with handheld ECG alone; nine patients at the time of their first handheld ECG recording, nineteen patients after 28 days of intermittent handheld ECG recording. Follow-up showed that of nine patients discovered at day one six had persistent AF and three paroxysmal AF. One additional patient who continued with registrations after day 28 had one AF episode at day 38. As this was not detected within 28 days he was not counted as newly diagnosed AF within the study framework. Seven patients showing episodes of repetitive irregular supraventricular extrasystoles (SVES) less than 10 seconds on handheld registrations were diagnosed with AF after an additional 24-hour Holter ECG.

Eighty percent of patients with detected AF were aged ≥ 65 years. The AF detection rate for patients< 65 years was 2.9% and for patients ≥ 65 years 4.1% (Pearson Chi Square test, P = 0.404). The patients in this study had in general low CHADS_2_ scores with a median of 2 (range 1–5). Slightly higher detection rates for AF could be seen among those with CHADS_2_ ≥ 2 compared to those with a score of 1, but no significant differences were seen (Pearson Chi Square test, P = 0.351). More male than female patients were discovered (4.6% and 3.0% respectively), but this difference was not significant (Pearson Chi Square test, P = 0.218).

In AF patients AF was recorded in 33.7% of registrations on average (SD ± 41.1 and range 1.3%–100%). Characteristics of AF registrations are displayed in Table 2.

**Table 2:** AF characteristics of newly diagnosed AF patients


**Paroxysmal AF, n (%)**		29	(82.9%)	
**Persistent AF, n (%)**		6	(17.1%)	
**Atrial fibrillation, n (%)**		31	(88.6%)	
**Atrial flutter, n (%)**		4	(11.4%)	
**Time to AF detection with Handheld ECG (n = 28)**				
	**Day 1**	9	(32.1%)	
	**Day 2–14, n (%)**	14	(50.0%)	
	**Day 15–28, n (%)**	5	(17.9%)	
**Diagnosis after additional Holter, n**		7		
		***Mean***	***SD***	***Range***
**AF registration (% of total registration time)**		33.7%	± 41.1	1.3%–100%
**AF at morning registration (% of AF registrations)**		47.7%	± 34.5	0%–100%
**Symptoms registered at AF episode (% of AF registrations)**		12.4%	± 28.7	0%–100%
**Time to first AF episode (days)**		7.3	± 7.6	1–28
**Mean heart rate during AF episodes (beats/minute)**		105	± 26.9	60–160

**Figure 4:**
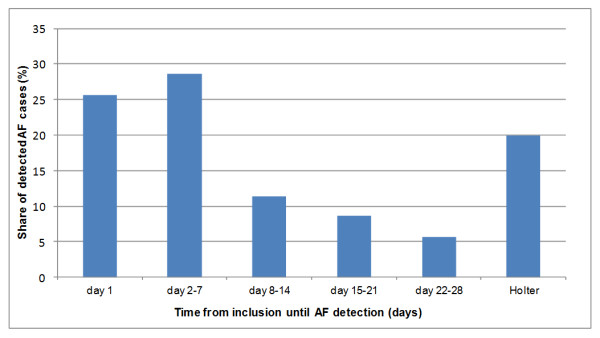
Time from inclusion until AF detection.

There was no difference regarding whether AF occurred in the morning or the evening. Only 12.4% of AF registrations were related to symptoms. On average AF was diagnosed after 7.3 days (SD ± 7.6; range 1–28). (Time to detection is illustrated in Figure 4). More than eighty percent of AF patients discovered with handheld ECG were found within 14 days. Six patients had a persistent AF. Four patients had atrial flutter. In case of typical p-wave morphology, as in atrial flutter, diagnosis was confirmed with 12-lead ECG or Holter ECG.

### Additional holter ECG investigation

Thirty additional Holter investigations were done. Twenty-four Holter recordings were made because of suspected AF on handheld ECG and resulted in seven more AF diagnoses. Four of these patients had episodes of more than 30 seconds on Holter; three had episodes of more than 10 seconds but less than 30 seconds. Six Holter investigations were done because of suspected brady-arrhythmias. Five patients received a pacemaker because of diagnosis of AV block II-III or sinus arrest. All five patients had a CHADS_2_ of 2, mainly hypertension and age ≥ 75. Three of them had ischemic heart disease. Their average age was 74.4 years with a range of 58–85.

## Discussion

Opportunistic screening in an out-of-hospital population with at least one risk factor for stroke (CHADS_2_) resulted in detection of 3.8% previously unknown AF, higher than in previous studies of out-of-hospital populations [21-23]. These results, if replicated in other studies, show the possibilities for the use of intermittent ECG registration as a screening instrument for detection of AF. A detection rate of 3.8% newly diagnosed AF is high, especially when considering that the study population was relatively young and healthy with few CHADS_2_ risk factors. AF patients were on average slightly older and had slightly higher CHADS_2_ risk factors than non-AF patients, but no statistically significant clinical differences were seen.

A survey of previously published studies did not reveal data comparing the efficiency of intermittent ECG recording with other screening methods for the detection of AF in an out-of-hospital population. A British multi-centre randomized controlled trial using systematic and/or opportunistic screening with pulse control and 12-lead ECG to detect AF among people over 65 years found incidences of less than 2% newly diagnosed AF for all methods (even when including patients detected outside the screening programme), within a year of screening [21,22]. Another study screening of a general practice population using pulse assessment found about 1% previously unknown AF [23]. Studies of post-stroke patients estimated a prevalence of 3.8–8.4% previously undiagnosed AF using 24-hour Holter and Event loop recorder ECG [17-19]. A recent Swedish study shows that intermittent handheld ECG recording compared with 24-hour Holter ECG substantially improves the detection of silent paroxysmal atrial fibrillation (AF) in post-stroke patients [20]. Continuous monitoring during a follow-up of 1.1 ± 0.7 years with an implanted device resulted in detection of 30% previously unknown AF, in a population of patients with risk factors for stroke, who had recently received a pacemaker, implantable cardioverter defibrillator or cardiac resynchronization therapy device [26]. Using such implantable devices for screening in large out-of-hospital populations is at present not suitable for economic reasons. Other devices, e.g. patch-based appliances with the possibility of long-term continuous recording, could however become an alternative for screening in large out-of-hospital populations [27].

The study design does not allow for any assessment of how representative the population is. The detection rate of AF, mean age and mean CHADS_2_ were comparable at all ten centres and this together with the size of the study population, consecutive inclusion, a very high participation rate and low drop-out increases the likeliness of representativeness.

### Device and method

The novelty in this study compared to other studies screening for AF is both the device itself and the screening method of prolonged intermittent recording for four weeks, both regularly and when having symptoms.

Handheld ECG in combination with the chosen screening method has advantages compared to other, traditional screening instruments. The device is small and involves almost no limitation to the mobility of the patients. Registrations are easy to perform and compliance is high, as supported by the results of the present study where 95% of all included patients had sufficient technical recordings. The ECG recording can immediately be transmitted to a website and assessed directly. Since there was no comparison group, cost-effectiveness cannot be addressed. The cost per screened patient using this method of screening is less than €100.

### Future screening: high compliance; shorter screening period?

The high compliance rate using this method indicates that it is feasible as a screening instrument for AF. More than 80% of AF diagnoses with handheld ECG were found within 14 days, suggesting that the registration period can be shortened without losing too much information. A major screening study has been launched in which 25 000 Swedes aged 75 and 76 years are randomized either to participate in a 14-day screening programme using 30-second handheld intermittent ECG recording to detect asymptomatic AF, or to act as control group [28].

### Detection of patients with conduction system disease

Six Holter investigations were done because of suspected brady-arrhythmias and five patients received a pacemaker because of diagnosis of AV block II-III or sinus arrest. All five patients were symptomatic, reporting dizziness and syncope but had not contacted healthcare previously. They were on average slightly older, had higher CHADS_2_ scores and significantly more ischemic heart disease (P = 0.024) compared to patients not receiving a pacemaker.

### Limitations

One quarter of the new AF cases were identified at the first registration, so they would have been detected with routine care involving a pulse check and/or ECG. The additional value of the handheld ECG with intermittent registrations is therefore in the detection of the remaining patients.

Seven out of 35 AF patients were confirmed only after an additional 24-hour Holter ECG. This makes it more difficult to say how many patients would have been found after a shorter screening of two weeks (decisions about doing an additional Holter were only made after the whole four-week period). Nevertheless only 24 Holter recordings were needed to find seven more AF cases, which makes this kind of intermittent screening still very efficient.

We used registrations of only 10 seconds due to the limited capacity of the handheld device; we do not know at present whether 30-second registrations of AF, as used in guidelines, are more relevant. The AF definition used by the European Society of Cardiology in its Guidelines from 2010 – ‘Any arrhythmia that has the ECG characteristics of AF and lasts sufficiently long for a 12-lead ECG to be recorded, or at least 30 s on a rhythm strip, should be considered as AF’ – is not empirical, but based on consensus [9]. Standard 12-lead ECG is a 10-second strip and diagnosis of AF based on a 10-second 12-lead resting ECG is used in other recent studies [6].

Providing lifelong anticoagulation to someone with a single 10-second episode of AF is definitely a leap too far at this stage. The patients in this study, however, had AF on more than 30% of their registrations on average (almost three minutes in a total registration time of only eight and a half minutes). Only AF registrations that lasted the full 10 seconds were counted as AF. How long these episodes lasted in reality is impossible to say. Two patients had a single 10-second episode of AF, which could not be reproduced during follow-up. It is therefore important in future screening projects that patients complete the screening period even if AF is diagnosed and further confirmatory testing will be required in many patients.

A disadvantage of the handheld ECG device is that only lead I is recorded, which sometimes makes it difficult to distinguish (1:1 and 2:1 blocked) atrial flutter from a regular supraventricular re-entry tachycardia such as AV-nodal re-entry tachycardia, which might have resulted in underdetection of atrial flutter [25]. At the same time, we cannot exclude the possibility of some cases with short runs of atrial tachycardia being mislabelled as AF because of the short 10-second recording time [25]. Ideally, this study should have been performed along with a more continuous monitoring device to help assess the accuracy of the outcomes.

## Conclusions

Opportunistic screening with intermittent handheld ECG registration over four weeks showed a detection rate of 3.8% of previously undiagnosed AF, in a population (n = 928) of relatively healthy and relatively young, out-of-hospital patients having at least one additional risk factor for stroke. This study shows very high compliance suggesting that opportunistic screening using this method could be a feasible technique for detection of AF.

## Competing interests

The authors declare that they have no competing interests. Neither Dr. Tijn Hendrikx, Dr. Rolf Hörnsten, Prof. Mårten Rosenqvist nor Dr. Herbert Sandström has any commercial interests in Zenicor Medical Systems AB.

## Authors’ contributions

TH took part in conceiving and designing the research, acquiring data, analysed and interpreted data, performed statistical analysis and drafted and revised the paper. He is guarantor. RH took part in acquiring data, analysing and interpreting data and revising the manuscript. HS took part in conceiving and designing the research, handled funding and supervision and made critical revisions of the manuscript. MR conceived and designed the research, handled funding and supervision and made critical revisions of the manuscript. All authors read and approved the final manuscript.

## Pre-publication history

The pre-publication history for this paper can be accessed here:

http://www.biomedcentral.com/1471-2261/13/41/prepub
